# *Triatoma venosa* and *Panstrongylus geniculatus* challenge the certification of interruption of vectorial *Trypanosoma cruzi* transmission by *Rhodnius prolixus* in eastern Colombia

**DOI:** 10.1371/journal.pntd.0012822

**Published:** 2025-01-27

**Authors:** Omar Cantillo-Barraza, Lídia Gual-González, Natalia Velásquez-Ortiz, Manuel Alfonso Medina Camargo, Paola González, Lissa Cruz-Saavedra, Adriana Castillo, Sara Zuluaga, Giovanny Herrera, Hanson Cowan, Andrés Velez-Mira, Luz Helena Patiño, Juan David Ramírez, Omar Triana, Melissa S. Nolan

**Affiliations:** 1 Grupo Biología y Control de Enfermedades Infecciosas, Universidad de Antioquia, Medellín, Antioquia, Colombia; 2 Centro de Investigaciones en Microbiología y Biotecnología—UR (CIMBIUR), Facultad de Ciencias Naturales, Universidad del Rosario, Bogotá, Distrito Capital, Colombia; 3 Department of Epidemiology and Biostatistics, Arnold School of Public Health, University of South Carolina, Columbia, South Carolina, United States of America; 4 Programa de Control de Vectores, Secretaría de Salud de Boyacá, Tunja, Boyacá, Colombia; 5 Unidad de Ecoepidemiología (PECET), Universidad de Antioquia, Medellín, Antioquia, Colombia; 6 Department of Pathology, Molecular and Cell-based Medicine, Icahn School of Medicine at Mount Sinai, New York, New York, United States of America; Tulane University School of Public Health and Tropical Medicine, UNITED STATES OF AMERICA

## Abstract

Reactivation of *Trypanosoma cruzi* transmission by native vectors with different domiciliation capabilities is a major concern for Chagas disease control programs*. T. cruzi* transmission via intra-domestic *Rhodnius prolixus* was certified as interrupted by the Pan American Health Organization in Miraflores municipality (Boyacá, Colombia) in 2019. However, *Triatoma venosa,* a native vector infected with *T. cruzi* has been increasingly found inside human dwellings across rural areas. In this study, the aim was to describe the eco-epidemiological aspects of *T. cruzi* transmission in the rural area of Miraflores. For this, we designed a comprehensive, multi-faceted study in 6 rural villages and performed: (*i*) A cross-sectional serological and molecular study enrolling 155 people and 58 domestic dogs living within 80 households, (ii) a domestic entomological survey, (iii) a determination of the natural infection and blood meal source in collected triatomine bugs, and (*iv*) an evaluation of synanthropic mammal infection by parasitological and molecular tools. The *T. cruzi* seroprevalence rates in humans and dogs were 9.03% (14/155) and 22.4% (13/58), respectively. Most infected humans were adults between the ages of 55 and 85 years old. No evidence of *T. cruzi* DNA was found using qPCR in human blood samples, but we found high parasitemia levels in the infected dogs. In total, 38 triatomine bugs were collected inside dwellings and peridomestic areas: 68.4% (26/38) *Triatoma venosa,* 29% (11/38) *Panstrongylus geniculatus,* and 2.6% (1/38) *P. rufotuberculatus.* Natural infection prevalence was 88% (22/25) for *T. venosa*, 100% (12/12) for *P. geniculatus,* and 100% (1/1) *P. rufotuberculatus*: only TcI was found. No evidence of *R. prolixus* was found in the area. Two feeding sources were identified in *T. venosa* (humans and cats), while *P. geniculatus* fed on cows and bats. Lastly, seven *D. marsupialis* were captured in peridomestic areas, three were infected with *T. cruzi* (TcI). The results suggest the existence of *T. cruzi* transmission cycle between triatomines, dogs, and opossums representing a risk of infection for the human population in rural areas of Miraflores. Despite PAHO declaring Miraflores municipality, Colombia an area of *T. cruzi* transmission interruption in 2019, this study documents evidence of a secondary vector establishing in domestic settings. *T. venosa* entomological surveillance is warranted to evaluate prospective human transmission risk in an otherwise ‘no-risk’ perceived Chagas disease region.

## Author summary

A major concern for Chagas disease control programs is transmission reactivation by native vectors that are not commonly found living within the domestic environment. In Colombia, transmission by primary vector species was certified as interrupted in Miraflores municipality in 2019, however other secondary native species have been found entering human dwellings in rural areas, emphasizing the need for entomological surveillance. In this study we collected and analyzed human and animal blood samples, and secondary vector species from six rural villages to detect *Trypanosoma cruzi* infections. The results show evidence of active infection among three native vector species, domestic canines, and peridomestic opossums. Our results are important, as vector species ecology continues to adapt warranting increased entomological surveillance to assess human transmission risk in an area perceived as free of Chagas disease transmission.

## Introduction

Chagas disease (CD) is a zoonosis caused by the protozoan *Trypanosoma cruzi*, transmitted to humans mainly by insects of the Triatominae subfamily (Hemiptera: Reduviidae) through stercorarian transmission [1]. In Latin America, more than 150 species of Triatomine bugs have been reported, yet three species: *Triatoma infestans, Triatoma dimidiata,* and *Rhodnius prolixus*, are responsible for the majority of vectorial transmission cases across the continent [2]. In Colombia, 26 triatomine species have been reported, 16 of which have been found naturally infected with *T. cruzi* [3,4]. Knowledge of underlying entomologic profiles is critical to tailoring effective vector control programs, particularly for Chagas disease whose initiatives are largely under-resourced [5,6].

Triatomine vectors have been historically targeted by some international initiatives including INCOSUR (Initiative of the Southern Cone Countries), IPCAM (The Initiative of the Central American Countries and Mexico), and IPA (Initiative of the Andean Countries) to control Chagas disease in South, Central and North America [7]. In 2011, 6 countries from IPCAM certified the elimination of *R. prolixus* [8,9], but recently the same species was found again infesting houses and peridomicile structures in Mexico [10]. Moreover, in IPCAM regions, other species can take over the niche left by previous species, as is the case of *T. dimidiata* in areas once infected by *R. prolixus* [7,11–13]. For the INCOSUR countries, they did extensive insecticide-spraying to achieve the elimination of *T. infestans* [14,15]. However, residual foci of *T. infestans* were later found in Brazil [16–19], some (re)infestation processes in Peru related to external sources [20,21], and infestations by other native species in different INCOSUR territories [22,23]. Therefore, the main challenges for these new scenarios in the different initiatives are: efforts to target other native species and insecticide-resistant populations, further research, and fund long-term sustainable surveillance systems [7].

Following the elimination programs model from INCOSUR for *T. infestans,* Colombia prioritized the elimination of domestic *R. prolixus* due to its epidemiological relevance, wide distribution, and strictly domiciliated population presence [24]. Boyacá department, located in the eastern Andean region, is one of the most endemic departments for Chagas disease [25]. Historically, government institutions have implemented control and prevention programs to eliminate intra-domestic *R. prolixus* in this region. As a result, Boyacá has 24 municipalities certified free from *R. prolixus-*mediated intradomicile *T. cruzi* transmission, making it the department with the largest number of certified *T. cruzi*-free cities in Colombia [26–28]. However, nine triatomine species have been described in Boyacá, five of which (*T. dimidiata, Triatoma venosa, Panstrongylus geniculatus, Panstrongylus rufotuberculatus,* and *Rhodnius pictipes*) have recently been identified as infected with *T. cruzi* [29]. Similar epidemiological situations have been described in other countries with successful *T. infestans* entomological control where the reinfestation of native vectors, such as *Triatoma brasiliensis,* among others, maintain *T. cruzi* transmission risk post-primary vector elimination [7]. Several studies in Boyacá evaluating the ecological, molecular, and spatial characteristics of triatomine fauna post-certification have shown that vector species such as *T. dimidiata* and *T. venosa* are the most abundant native vectors locally. Both species have the greatest epidemiological importance due to the household infestation levels in the northeast and southwest respectively [26,30,31]. Moreover, these vector species have high *T. cruzi* infection levels and often feed on humans and domestic animals [26,30]. These ecological characteristics highlight the need for serological studies to obtain a close look at the CD exposure risk among people in areas where the main transmitter species is controlled [26,30].

The Miraflores municipality, located in the Southwest of Boyacá, is a municipality certified by PAHO as free of *T. cruzi* transmission by intradomestic *R. prolixus* since 2019 [28]. Nevertheless, a recent study conducted in this municipality showed relevant infestation and colonization index of 14.5% and 16.4% respectively and high *T. cruzi* infection levels among *T. venosa* collected from rural homes [29]. The occurrence of *T. venosa* in households and its possible involvement with domestic animals and humans in the transmission cycle of *T. cruzi* creates concern among residents and constitutes a public health threat [26,29]. Therefore, this study aimed to describe the eco-epidemiological characteristics of *T. cruzi* transmission in a rural area of Miraflores municipality through (*i*) a cross-sectional evaluation of *T. cruzi* infection among human and dog populations using serological and molecular tools(ii) entomological surveys(iii) natural *T. cruzi* infection and blood meal source determination in triatomines, and (*iv*) evaluation of *T. cruzi* infection in synanthropic mammals.

## Materials and methods

### Ethics statement for human and animal evaluations

The Committee of Ethics in Research (meeting of Nov. 16, 2021) and the Committee for Animal Experimentation (meeting of Dec. 9, 2021) of the Universidad de Antioquia approved human and animal participation in this study, respectively. All adult participants (aged ≥18 years) signed the informed consent form. Children (<18 years old) were enrolled in the study after their parents or guardians signed the informed consent on their behalf. All animals were handled in strict accordance with good animal welfare, as defined by the Colombian Code of Practice for the Care and Use of Animals for Scientific Purposes, established by Law 84 of 1989.

### Study area

**Fig 1:**
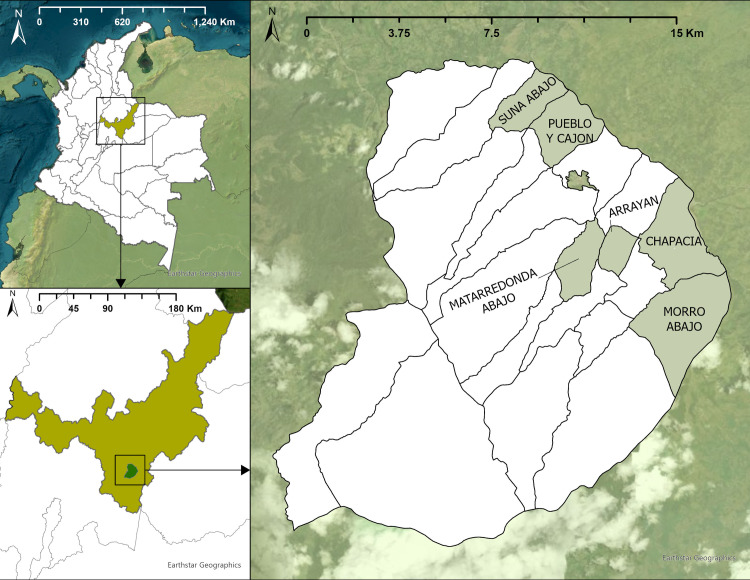
Geographic location of the study area. Top left: Boyacá Department, Colombia. Bottom left: Miraflores municipality, Boyacá Department. Right: Rural villages selected for the study: Arrayán, Chapacía, Matarredonda Abajo, Morro Abajo, Pueblo y Cajón, and Suna Abajo. The map was generated using ArcGIS Pro 3.1.2 (ESRI, Redlands, CA, USA). Basemap is obtained from the ESRI basemap repository Earthstar Geographics Imagery (https://services.arcgisonline.com/ArcGIS/rest/services/World_Imagery/MapServer). The country, state, and municipality layers were downloaded from the ESRI’s Catalog ArcGIS Online, created by the Instituto Geográfico Agustín Codazzi (IGAC), under open standard use license “Creative Commons CC BY 4.0”, with extension West: −81° 46' 0.5334", East: −66° 41' 7.4724", North: 13° 32' 4.5456", South: −4° 13' 55.128" (https://www.arcgis.com/home/item.html?id=07262475c7b0435d8978c8298b3ecd61). The country boundaries was obtained from the ArGIS Online database under public domain (https://www.arcgis.com/home/item.html?id=07262475c7b0435d8978c8298b3ecd61).

This study was conducted in Miraflores municipality (5° 11' 43.46" N; 73° 8' 39.55" W), located in the Department of Boyacá in the Andean Region of Colombia (Fig 1). It is a tropical sub-humid region with an annual rainfall of 1300 mm and an annual average temperature of 15.5°C. Rainy seasons are bimodal extending from April to June and August to November. The dry season extends from December to March. The study area is mostly livestock production and crops such as coffee, sugar cane, passion fruit, and pitahaya, among others.

### Study design and sample size

This cross-sectional study was conducted from December 2021 to December 2022. Six villages were selected for convenience based on accessibility, closeness to the urban center, and previous reports of triatomine bugs [29]. Six rural villages were selected: Arrayán, Chapacía, Matarredonda Abajo, Morro Abajo, Pueblo y Cajón, and Suna Abajo (Fig 1 and Table 1). The inclusion criteria for human sample collections were persons of all ages residing in the study-selected villages who agreed to participate and signed the informed consent. Participants were recruited through house-to-house visits to each village. To obtain the sampling frame, a population census was conducted in the six villages, registering information about the number of inhabited houses, number of people, and presence of domestic animals. A total of 283 households inhabited by 1458 people were registered in the census. Appropriate and representative sample size (n) estimates for the human survey were calculated using the Epi-info V 2000 software with the priors: 3% confidence level, 2.6% infection frequency (according to Suescún) and 3% error [32]. These calculations yielded a necessary sample size of 101 people. Probabilistic sampling was stratified by household within each village, using a probability proportional to size, 118 households were surveyed, 80 of which human and/or canine biological samples were collected. Convenience sampling was used at household level to obtain biological samples, whereas household survey and triatomine searching was performed in all the households. Household-level surveys provided information about resident sightings of wild animals inside or around the house and property, and areas where they carry out domestic activities (washing clothes, cooking food, raising domestic fowl, and growing crops, with a maximum radius of 30 meters from the house).

**Table 1:** Results for the blood meal analysis, parasite load and number of *T. cruzi* positive individuals for each blood meal source and triatomine species.

Triatomine species	Blood meal source species	Number of positive triatomines (%)	Parasite load range pe/mL (min–max)
*Triatoma venosa (n = 26)*	*Homo sapiens*	10 (38.5)	365–1,784,826
	*Felis catus*	1 (3.8)	4,312
	ND	15 (57.7)	319–82,563
*Panstrongylus geniculatus (n = 11)*	*Bos taurus*	1 (9.0)	106,677
	*Molossus barnesi*	1 (9.0)	145,746,800
	ND	9 (81.8)	12,441–196,175,888
*Panstrongylus rufotuberculatus (n = 1)*	ND	1 (100)	163,841,728

ND = Not determined for triatomines that did not have a recent bloodmeal.

### Triatomine collection and processing

Three entomological surveys were conducted at each study location from December 2021 to December 2022. All procedures were performed by Boyacá department health service (BDHS) technicians following the National Protocols of Entomological Surveillance [33]. Triatomines were searched in 80 households, indoors (intradomicile) and outdoors (peridomicile) for 30 min following the same protocol using a flashlight to see into wall cracks and crevices, behind wall-mounted picture frames, behind furniture, inside closets, and especially under bedding material. Triatomines were transported to the laboratory, registered, and identified using taxonomic keys [34].

All collected triatomines were evaluated for *T. cruzi* infection using parasitological and molecular methods at the Universidad de Antioquia, Medellín, Colombia. Feces were obtained by abdominal compression, diluted in 300 μL of sterile phosphate buffer saline (PBS) (pH: 7.2), and used for *T. cruzi* DNA extraction. Genomic DNA was extracted from 200 µL of feces using a DNeasy Blood & Tissue Kit (Qiagen, Germantown, MD, USA) following the manufacturer’s instructions.

### Serological survey in humans and canines

#### Humans.

Approximately 5 mL of whole blood was collected in vacutainer tubes via venipuncture, and each sample was subjected to serological and molecular tests. An aliquot of 200 μL was stored in a K3-EDTA solution for molecular diagnostics. To obtain serum, 3 mL of the sample was centrifuged for 15 minutes at 1000 g, the supernatant was then placed into a new 1.5 mL tube, which was stored at −20°C until processing. All patients were evaluated using two enzyme-linked immunosorbent assay (ELISA) tests with different principles, according to the recommendations made by the National Institute of Health, Colombia: a total antigens commercial ELISA (ELISA Chagatest Winner) and a recombinant antigens ELISA (CHAGATEK ELISA, Microelisa system) following the manufacturer’s instructions. The kit uses a cut-off value calculated from the average optical density (OD) value between the negative controls and adding 0.2 to determine the positive values. The incongruent samples were analyzed using a third additional serological tests: indirect immunofluorescence assay (IFA), in which the incongruent samples with titers of 1:40 or more were considered positive. The subjects with positive results were sent to the regional hospital for further assessment, follow-up, and consideration of possible treatment according to the National Chagas Departmental Program.

#### Canine host reservoir.

Dogs were selected from the participating households, where their owners were interested in animal testing. The inclusion criteria for these mammals were as follows: (I) born and raised in the study area, (II) having a recognizable owner, and (III) informed consent from their owners. For each animal, two 5 mL radial vein blood samples were collected using serum and EDTA vacutainers and stored at 4°C until processed. For serum processing, samples were centrifuged at 5000 × g for 10 min, and the extracted serum was stored at −20°C until diagnostic assays were performed.

Two serological tests were performed to detect anti-*T. cruzi* IgG. All samples were subjected to one initial screening by an ELISA homemade test, using total protein extract as antigens, prepared from *T. cruzi* isolates (I.RHO/CO/00/CAS-15.CAS; I.TRI/CO/03/MG-8.MAG). Previously confirmed positive and negative samples were used as control values to determine OD limits for seropositivity and seronegativity. OD values higher than 2 standard deviations (SD) of the average OD for negative controls were considered seropositive. All ELISA-positive samples were confirmed by an immunofluorescence antibody test (IFAT) with a titer of 1:40 as a positive cut-off. Samples that were seropositive for both tests were considered to be seropositive for *T. cruzi* [35].

### Wild host survey and small mammal *Trypanosoma cruzi* detection

Small mammals were captured using 10 baited traps (5 Tomahawk and 5 Sherman) containing a mixture of peanuts, bananas, oats, and fish. At each village, ten traps were located for three nights in peridomestic areas, including permanent or temporal structures built and used by humans or their domestic animals. Traps were distributed in linear transects, with captured points established 20 m apart from each other. Wild mammals were anesthetized intramuscularly (9:1 ketamine hydrochloride 10%, and xylazine 2%), according to Roque et al [36]. Blood samples were collected and stored for DNA extraction and molecular analysis. Also, two tubes containing NNN medium covered with a LIT overlay were inoculated with 0.3 to 0.6 mL of blood. Tubes were examined weekly for the presence of *Trypanosoma spp*. appearances for three months.

### Molecular analysis for triatomine, human, and mammal samples

All collected triatomines, domestic dog samples from seropositive humans, and *Didelphis marsupialis* whole blood samples and positive hemocultures were screened for *T. cruzi* using qualitative real-time PCR (qPCR) targeting satellite DNA [37]. Human DNA was extracted from 200 µL of EDTA whole blood using DNeasy Blood & Tissue Kit (Qiagen, Germantown, MD, USA) according to the manufacturer’s instructions. Total DNA was diluted with 100 µL elution buffer and stored at −20°C until molecular diagnosis. The PCR was performed in 25 µL final volume containing 40–50 ng of genomic DNA, 1X of buffer, dNTP 0.04 mM, MgCl_2_ 1.5 mM, 0.4 µM of each primer (TCZ1 and TCZ2), and 0.05 U of Taq DNA polymerase (Invitrogen, Carlsbad, CA, USA). The thermal cycling conditions were as follows: pre-heating at 95°C for 15 min, followed by 40 cycles at 95°C for 10 s, 55°C for 15 s, and 72°C for 10 s in a thermocycler [38].

Positive *T. cruzi* samples were analyzed for molecular discrimination between *T. cruzi* discrete typing units (DTUs) based on spliced leader intergenic region (SL-IR) gene amplification using the primers TCC, TC1, and TC2, as previously reported [39]. The second PCR was performed in 25 µL final volume containing 40–50 ng of genomic DNA, 1X of buffer, dNTP 0.25 mM, 2 mM MgCl_2_, 0.4 µM of each primer, and 0.05 U of Taq DNA polymerase (Invitrogen). Thermal cycling conditions were as follows: pre-heating at 94°C for 5 min, 35 cycles at 94°C for 30 s, 55°C for 30 s, and 72°C for 45 s, and a final extension at 72 °C for 5 min, in a thermocycler. Amplification products were run on a 1.5% agarose gel stained with ethidium bromide and visualized under UV light.

### *T. cruzi* quantification, standard calibration, limit of detection, and parasite burden in triatomine, humans, and canines

An evaluated and validated qPCR protocol for *T. cruzi* quantification was used for seropositive humans and domestic dog samples developed by Ramírez et al 2015 [40]. For field-collected triatomines, a standardized qPCR protocol was used including standard calibration, limit of detection (LOD), and parasite burden reported by Velásquez-Ortiz et. al 2022 [30]. *T. cruzi* MHOM/CO/04/MG strain (TcI) metacyclic trypomastigotes were used as previously reported. The triatomines were isolated and quantified to obtain 10^11^ parasites per mL. QPCR reagents included a TaqMan Fast Advanced Master Mix 2× (Roche Diagnostics GmbH, Mannheim, Germany), water, and primers cruzi1 (0.75 μM) (5′-AST CGG CTG ATC GTT TTC-3′), cruzi2 (0.75 μM) (5′-AAT TCC TCC AAG CAG CGG ATA-3′) and a cruzi3 probe (0.05 μM) (FAM-CAC ACA CTG GAC ACC AA-NFQ-MGB), targeting the satellite DNA (166 bp) [38]. The reaction conditions were 50°C for 2 minutes, 95°C for 10 minutes, and 40 cycles at 95°C for 15 seconds and 58°C for 1 minute. The nuclear gene 28S rDNA was used as internal amplification control by qPCR using the primers D2F (5’- GCGAGTCGTGTTGCTTGA TAGTGCAG- 3’) and D2R (5’-TTGGTCCGTGTTTCAAGACGGG- 3’) following the conditions described before [41]. Real-Time Software Quant Studio Design and Analysis (Applied Biosystems, Waltham, MA, USA) was used to automatically calculate the number of parasites per sample, using the linear regression equations obtained from the standard curve.

### Triatomine bloodmeal source(s)

Triatomine bloodmeal sources were assessed using vertebrate 12S rRNA gene PCR targets, and obtained through amplification of a 215 bp fragment using primers L1085 (5′-CCCAAACTGGGATTAGATACCC-3′) and H1259 (5′-GTTTGCTGAAGATGGCGGTA-3′) [42]. Electrophoresis was performed on a 1.5% agarose gel stained with SYBR Safe and visualized under UV light into a molecular imager Gel DOCTM XR+ with Image Lab software (Bio-Rad Laboratories Inc., California, USA). The resulting sequences in both directions were edited in MEGA X software, assembled, and manually checked in all base changes according to peak quality (height, no overlapping, and evenly spaced). The sequences were later submitted to BLAST (https://blast.ncbi.nlm.nih.gov) for similarity search defining each species with a percent identity higher than 98% and an e-value close to 0.00.

### Statistical analysis and geospatial approaches

Risk factors associated with domiciliation, defined as the presence of adults and/or nymphs inside the dwellings or peridomicile structures [43]. Entomological indices were determined according to the following calculations as described by the WHO: 1) the infestation index: (the number of houses infested by triatomines/number of houses examined)×100; 2) the colonization index: (houses with triatomine nymphs/number of houses positive for triatomine)×100; 3) the density index: (number of triatomines captured/number of houses examined); 4) the crowding index: (number of triatomines captured/number of houses with triatomines); and 5) natural infection index: (number of infected pools/total number of analyzed pools)×100. The standardized national cross-sectional survey of Chagas disease risk in Colombia was employed to evaluate known associated risk factors [44]. The questionnaire pinpoints different household risk factors including house construction materials, peridomicile characteristics, and animal presence in the household. Proportional comparisons were performed across seropositive values for sex and age using Chi-square test with a significance level below 0.05. Univariable Poisson regression analyses were performed to evaluate survey household characteristics associated with the number of triatomines collected at each location. Data analysis was performed using R Studio, R version 4.3.1 (Free Software Foundation, Boston, MA, USA).

Base maps were extracted from the GADM database (www.gadm.org). Google Earth v.2.5 was used to determine the coordinates for all municipalities where triatomine insects and domestic dog samples were collected. The individual study location coordinates were captured using a handheld GPS (Global Positioning System) device. Coordinates were recorded in the WGS 84 Datum (World Geodetic System 1984) geodetic coordinate system. The geospatial visualization was performed in ArcGIS Pro 3.1.3 (ESRI, Redlands, CA, USA).

## Results

### Entomological survey, *T. cruzi* infection rate, and blood meal sources

**Fig 2:**
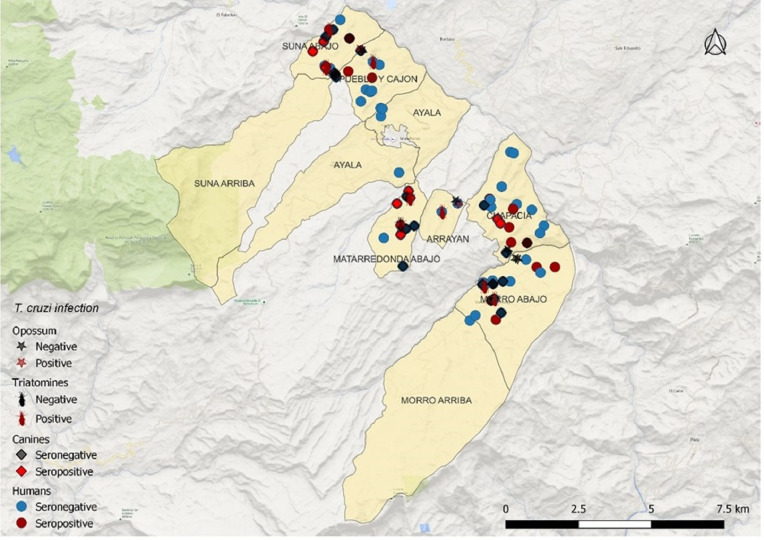
*Trypanosoma cruzi* infection distribution across geographical location, host, and vectors. The map was generated using Google Earth v.2.5 (https://earth.google.com/web) and the basemap for the municipality boundaries extracted from GADM (https://gadm.org/maps.html). The symbols for the triatomines were downloaded as a GIF file from BioRender (https://biorender.com) and embedded as vector in the maps using the household coordinates.

A total of 38 triatomine bugs were collected from the six villages evaluated (Table 1). Around 68.4% (26/38) of the triatomines collected were *T. venosa,* 29% (11/38) were *P. geniculatus,* and 2.6% (1/38) were *P. rufotuberculatus*. Among all triatomines, 76% (29/38) were collected intradomicile and 23.6% (9/38) in peridomestic habitats (household surroundings including chicken coops, barn, wood piles, and rock piles). Ten collected nymphs of *T. venosa* were collected intradomicile in Matarredonda Abajo. Triatomine species were heterogeneously distributed: *T. venosa* were located in Arrayán, Chapacia, and Mataredonda Abajo; *P. geniculatus* Morro Abajo, Pueblo y Cajón, and Suna Abajo; and *P. rufotuberculatus* in Pueblo y Cajón (Fig 2). *T. cruzi-*infected triatomine bugs were present in all six villages. The overall domicile infestation index was 11.2% (9/80), with a 33.3% (3/9) colonization index, 0.48 density index, and 4.22 crowding index. The highest infestation indices were observed in Suna Abajo 17.6% (3/17) and Matarredonda Abajo 16.6% (2/12), while the highest density index was found in Matarredonda Abajo (0.5). Among the captured *T. venosa* specimens, 88% (22/25) were positive for *T. cruzi* by qPCR. Moreover, 100% (12/12) of *P. geniculatus* and 100% (1/1) of *P. rufotuberculatus* were positive for *T. cruzi.* All the *T. cruzi* positive specimens were confirmed as TcI DTU. The median parasitic load among the positive triatomines was 28,774,446 pe/mL. *P. geniculatus* had the highest parasite load with a median of 57,406,337 pe/mL (range between 1,103 and 196,175,888 pe/mL) followed by *T. venosa* with a median parasitic load of 142,555 pe/mL (range between 319 and 1,784,826 pe/mL). Finally, the *P. rufotuberculatus* collected had a load of 163,841,728 pe/mL (Table 1). Four blood-meal sources were found among 13 triatomine bugs (10 *T. venosa* and 3 *P. geniculatus)*. *Homo sapiens* were the most frequent blood source for *Triatoma venosa*, suggesting humans as an important blood source for this triatomine species, followed by cats (*Felis catus*). Human blood meal was detected in the specimens collected indoors, while cat blood meal was detected in insects collected in the peridomicile. In Chapacía and Arrayán, humans were the only blood meal source identified for these triatomine species. Regarding *P. geniculatus*, blood meal sources identified were bulls (*Bos taurus*), and bats (*Molossus barnesi)* (Table 1). Cows were detected in peridomestic *P. geniculatus* specimens collected in Morro Abajo. There were no cow stables in the house where the specimens were collected, however, such structures were present in the neighboring houses. Additionally, a specimen collected in Pueblo y Cajón had a bat blood meal source.

The results of the univariable Poisson regression models are presented in S1 Table. There were significant positive associations in the unadjusted model between the number of triatomines collected and having tarnished and untarnished walls, roofs made out of mud tile, fiber cement tile, or wood, having dirt or wood floor, having a chicken coop, a stable, a porch, an oven outdoors, presence of wattle (roof storage compartment), wall cracks, and more lightbulbs in the outside of the property. The number of triatomines collected were also positively associated with owning domestic chickens, and rabbits, and having observed the presence of rodents, specifically around the house. The type of environment positively associated with the number of triatomines was forest surrounding the residences. Inversely, the number of triatomines collected was significantly negatively associated with the time living in the household, having partially tarnished walls, zinc roofs, having a barn outside the house, having wood piles around the house, owning cats, having seen opossums around the house, having presence of bushes, trees or epiphytic plants around the house, and living in an area surrounded by pasture fields.

### Seroprevalence of *T. cruzi* infection in humans

A total of 155 human serum samples were collected from participants ranging from 4 to 93 years of age, 82 (52.9%) were women and 73 (47.1%) were men (Table 2). The number of human subjects enrolled per house ranged from one to seven. A total of 14 patients were positive for two serological tests corresponding to an overall seroprevalence of 9.03% (95% CI: 5.0–13.8%). The seroreactivity by sex and villages is shown in Table 2. There were no significant differences between seropositivity rates and sex or age. No evidence of infection was found in patients under 14 years old and the highest seroprevalence was observed in those aged 70–80 years (21%). Further, *T. cruzi* DNA was not detected in seropositive human samples, suggesting chronic infection.

**Table 2:** *T. cruzi* infection and serological data for humans by sex and village.

Variable	Humans enrolled N = 155 (%)	Seropositive N = 14 (%)
**Age (Years ± SD)**	49.5 ± 23.1	68.3 ± 10.7
** ≤18 years**	21 (13.5)	0
** 19–29 years**	12 (7.7)	0
** 30–39 years**	20 (12.9)	0
** 40–49 years**	21 (13.5)	0
** 50–59 years**	24 (15.5)	4 (28.6)
** 60–69 years**	22 (14.2)	3 (21.4)
** ≥70 years**	36 (23.2)	7 (50.0)
**Sex**		
** Male**	73 (47.1)	6 (8.2)
** Female**	82 (52.9)	8 (9.8)
**Villages**		
** Chapacía**	36 (23.2)	4 (11.1)
** Morro Abajo**	18 (11.6)	4 (22.2)
** Suna Abajo**	44 (28.4)	5 (11.3)
** Matarredonda Abajo**	24 (15.5)	0
** Pueblo y Cajón**	23 (14.8)	1 (4.34)
** Arrayán**	10 (6.5)	0

Seropositive persons were identified in Chapacía, Morro Abajo, Pueblo y Cajón, and Suna Abajo. Morro Abajo, Suna Abajo and Chapacía presented the highest seroprevalence of 22.2% (4/18), 11.3% (5/44) and 11.1% (4/36), respectively. Pueblo y Cajón had a lower seroprevalence with 4.3% (1/23), with Arrayán and Matarredonda Abajo had no human infections (Table 2).

### Household characteristics

Of the 80 households evaluated, 17.5% had a seropositive resident, 11.3% (9/80) had triatomines inside or around the dwellings, and 22% reported having seen triatomines inside or around the household in the past year. Among the most important findings, the bedroom was identified as the most common indoor area with triatomine presence, brick walls were the most common material of walls, dogs were the most frequent domestic animal in the house, and rats and opossum *D. marsupialis* were the most common sighted sylvatic mammal. Other household and peridomestic characteristics are described in S2 Table.

### *T. cruzi* infection in dogs

A total of 58 domestic dogs were evaluated in this study. The mean and standard deviation age of the dogs was 5.04 ± 3.37 years (range between 6 months to 13 years) (Table 3). A total of 13 dogs (22.4%) were seropositive for *T. cruzi* by both ELISA and IFAT tests. Seropositive dogs were identified in Chapacía, Matarredonda Abajo, and Suna Abajo. The seropositive dogs were between 2 and 12 years old, 53.8% (7/13) were female, and 46.2% (6/13) were male (Table 3). Moreover, 61.5% (8/13) of the seropositive dogs had *T. cruzi* DNA presence in their blood. Only infected dogs from Mataredonda and Suna Abajo were PCR-positive. Finally, all the PCR-positive dogs were found infected with TcI with a median parasitic load of 358.3 pe/mL. All PCR-positive dogs were from different houses except two that were from the same house (S3 Table).

**Table 3:** *T. cruzi* infection and serological results among dogs by age, sex and village.

Variable	Dogs enrolled N = 58 (%)	Seropositive N = 13 (%)	PCR positives N = 8 (%)
**Age (Years ± SD)**	5.0 ± 3.4	5.8 ± 3.8	5.3 ± 3.7
** Puppy (3 m to ≤1 y)**	4 (6.9)	0 (0)	0 (0)
** Young (>1 to ≤4 y)**	29 (50)	6 (46.2)	6 (75.0)
** Adults (>4 y to ≤7 y)**	10 (17.2)	4(30.7)	1 (12.5)
** Geriatric (>7 y)**	15 (25.8)	3 (23.1)	1 (12.5)
**Sex**			
** Male**	38 (65.5)	7(53.8)	4 (50.0)
** Female**	20 (34.5)	6 (46.2)	4 (50.0)
**Villages**			
** Chapacía**	11 (20)	3 (23)	0 (0)
** Morro abajo**	11 (20)	0 (0)	0 (0)
** Suna Abajo**	21 (38.2)	5 (38.5)	4 (50.0)
** Matarredonda Abajo**	15 (27.7)	5 (38.5)	4 (50.0)

### *T. cruzi* infection in synanthropic mammals

Seven *D. marsupialis* individuals were captured in peridomestic areas of Arrayán, Chapacía, Matarredonda Abajo, and Pueblo y Cajón. Three *D. marsupialis* collected in Arrayán, Matarredonda Abajo, and Pueblo y Cajón had evidence of *T. cruzi* infection, and two had positive hemoculture assays from Matarredonda Abajo and Pueblo y Cajón. TcI was detected in positive specimens.

### Geospatial visualization

The geographical distribution of *T. cruzi* showed different distribution patterns: Matarredonda Abajo had a high infection index for *T. venosa* as well as PCR-positive dogs and one infected *D. marsupialis.* Similarly, in Chapacía there were infected *T. venosa*, human and canine seropositive samples, and infected *D. marsupialis*. On the other hand, Suna Abajo had high entomological indices for *P. geniculatus*, some of which were *T. cruzi-*positive, seropositive humans, and PCR-positive canines. Moreover, one household in Suna Abajo had positive triatomines, PCR positive dog, and a human seropositive, two households in Suna Abajo, one in Arrayán, and one in Pueblo y Cajón had positive triatomines and seropositive canines. Finally, in Pueblo y Cajón infected *P. geniculatus,* seropositive humans and canines, and positive *D. marsupialis* were identified (Fig 2). The data surface maps also enabled the identification of potential transmission hotspots, which are related to villages where infected human-fed triatomines, infected dogs, and positive synanthropic mammals overlap (Fig 2).

## Discussion

Domestic infestations and intrusive behavior of native vector species are the primary challenges in *T. cruzi* transmission control programs, once primary vector transmission by *T. infestans* and *R. prolixus* has been interrupted [7,45–47]. After the virtual elimination of *R. prolixus* in Boyacá Department, native species such as *T. dimidiata, T. venosa,* and *P. geniculatus* are emerging as secondary vectors maintaining the *T. cruzi* transmission cycle [26,30,31]. However, little research has been done on *T. cruzi* human transmission and renewed infection risk due to these new scenarios [22,35]. The results presented demonstrate the existence of *T. cruzi* transmission risk by native vectors and host mammal reservoirs in the Boyacá department, Colombia. No evidence was found to confirm human transmission, nonetheless, the current study discusses proof obtained regarding the existence of an active transmission cycle with intrusion in Miraflores that puts the population at risk of activation of vectorial transmission after *R. prolixus* elimination.

In the present study, a 9.0% *T. cruzi* seroprevalence was identified in humans in the rural area of Miraflores municipality. This is higher than the estimated seroprevalence reports for Colombia and other countries where previous studies showed seroprevalence between 2.5% and 7.8% [25,32,48,49]. Age differences in sampled populations could explain different seroprevalence rates. Another explanation for this discrepancy would be historical transmission reactivation, due to newly established peridomestic cycles by native and secondary vectors that have not been subject to vector control intervention. In Brazil, similar scenarios have been identified in areas where native species become more relevant due to increased presence in the domestic environment [50]. In this study, triatomines were more present in households reporting classic “hiding” spots such as tiled roofs, wood floors, or outdoor structures that provide shelter like chicken coop, stables, or wattle among others, that have been historically associated with triatomine presence [51]. The presence of triatomine-friendly refuges in the peridomiciles, offer a special challenge during vector control efforts, and false perception of effective methods can influence native vectors persisting in the household surroundings [52]. Minuzzi-Souza et al demonstrated that synanthropic triatomines maintain the *T. cruzi* transmission risk in regions with low surveillance efforts, highlighting the importance of the study findings [53]. Evidence of infected triatomine presence in the domicile and peridomiciles in this study requires further surveillance and control efforts, to reduce the likelihood of *T. cruzi* transmission reactivation in Miraflores.

While peridomestic *T. cruzi* transmission was noted, the present study did not identify evidence of *T. cruzi* transmission in human residents after intradomicile-*R. prolixus* vector elimination. No infection was found in residents younger than 14 years old, and seropositive samples were DNA-negative, suggesting the inexistence of acute infection. Similar findings were observed in Brazil, where Pavan et. al (2023) discussed that the lack of acute infection restricts the surveillance despite favorable conditions for the maintenance of *T. cruzi* transmission [48]. Nonetheless, the expansion of native vector species can extend *T. cruzi* transmission-risk areas, and the high seroprevalence rates among the older population in our study could be a consequence of cumulative vector-mediated exposure over their lifetime, which highlights the need for consistent epidemiological surveillance to identify future active transmission cases [46,54].

The epidemiological scenario described can be related to *R. prolixus* virtual elimination. Prior reports did not provide entomological indices on infestation and *T. cruzi* infection among other vectors, thus a false perception *T. cruzi* transmission elimination could have increased the risk reactivation [7,55,56]. According to Ferreira and colleagues, a correct interpretation of entomological indices could help determine *T. cruzi* transmission risk, as some vectors are less efficient in transmitting the parasite than others [57]. In Boyacá, *T. cruzi* transmission could be related to other epidemiologically relevant species such as *T. venosa* and *P. geniculatus.* In fact, there is evidence across Boyacá that emerging species show intrusive behavior after household chemical interventions [26,31]. A similar situation was reported in Brazil, where there is an overlapping distribution of infected domestic and sylvatic species (e.g., *T. petrocchiae* and *T. pseudomaculata* with *T. brasiliensis*) invading and/or colonizing human dwellings. These were found feeding on humans and domestic animals, thus increasing the risk of *T. cruzi* transmission, and potentially supporting the progressive adaptation of sylvatic species to anthropogenic environments [57–59].

Domestic dogs are considered a useful sentinel for many zoonotic diseases, and they are significant for Chagas disease epidemiology because their parasitemia may indicate *T. cruzi* transmission hotspots [36,60]. In this study, dogs had 22.4% seropositivity, which is higher than that reported in previous studies among dogs in Colombia: a 4.6% seroprevalence was reported in Boyacá, from one municipality with *T. dimidiata* infestation [31], whereas a 5% seroprevalence was found in Valledupar [61]. Conversely, studies with high *T. venosa* infestation found a 44.1% seropositivity with a 36.4% PCR-positivity in Boyacá, and a 27.9% seropositivity and 43.3% PCR-positivity in Santander [26,62]. Given the results obtained in dogs in Miraflores, the higher *T. cruzi* infection and parasite load could be evidence of active parasite transmission; especially in Suna Abajo where *T. cruzi* positive triatomines and a PCR-positive dog were found in the same household. Matarredonda Abajo and Suna Abajo villages should receive greater attention as actively infected dogs demonstrated presence of TcI, a parasitic DTU associated with negative cardiac effects [63,64].

*Rhodnius prolixus* was not found in the present study, which confirms its intradomicile elimination by past efforts [29]. However, *T. venosa*, considered a native vector with less epidemiological significance in Colombia, exhibits certain eco-epidemiological characteristics that allow the maintenance of *T. cruzi* transmission in the Tenza Valley area in Boyacá [26]. In the current investigation, this species was the most captured and the only one showing colonization capacity, and a higher *T. cruzi* infection rate (a 92% as opposed to a 13.9% in Tenza Valley) [26]. This scenario is concerning, as human transmission reactivation could appear soon if no surveillance and control efforts are provided in the area. The other two species found in this study, *P. geniculatus* and *P. rufotuberculatus*, presented a 100% infection rate—albeit fewer individuals were collected, limiting the inference of infection rate. The ecological importance of these two species should still be taken into consideration, as *P. geniculatus* is a vector frequently associated with oral outbreaks in Colombia (reporting up to 40% infection rates) [39,65], and *P. rufotuberculatus* despite its narrower distribution, has been found inside dwellings in other departments [27]. Given all the positive insects were identified as TcI, with high parasite loads, and the evidence of human blood meal, circulating in intra and peridomestic habitats, there is a potential increase in *T. cruzi* transmission risk for the residents in the study area.

No recent human transmission was identified, nevertheless, the geographical distribution of human seropositive cases, dogs, triatomines, and opossums yield priority areas for public health intervention. Cross-sectional surveys are an increasingly used tool for Chagas disease control, and randomly sampling villages with low surveillance capacity can allow for rapid identification and resource management in areas of great need [55]. Geographical areas with active or previous transmission were identified by combining serological and molecular tests identifying presence of vectors, domestic, and peridomestic hosts. Actively infected opossums and vectors were found in Matarredonda Abajo, Pueblo y Cajón, and Arrayán suggesting peridomestic transmission. Moreover, infected dogs were found in Suna Abajo and Matarredonda Abajo, along with infected vectors and high vector infestation rates, warranting the need for improved surveillance to identify potential transmission risk. High vector infestation rates positive for human bloodmeal were found in Suna Abajo and Matarredonda Abajo, both areas with PCR-positive canines, suggesting active domestic transmission. Lastly, Morro Abajo had seropositive humans and infected vectors, with evidence of human bloodmeal, suggesting potential transmission risk in the area. Based on the geospatial distribution of the results, the regions requiring priority intervention would be Matarredonda Abajo, Suna Abajo, Pueblo y Cajón, and Morro Abajo.

Some limitations in the present study should be acknowledged including (i) there were logistical challenges to carry out sampling effort for mammals for more than three days in all the dwellings in the study, which could have affected the non-capture of rodents; (ii) it was not possible to sample all the residential dogs in the households sampled, as oftentimes during the visit the dogs were not present in the house, or had escaped when attempted to recruit for sampling; (iii) given the role of the health workers from the Boyacá Health Department performing the collections, it was not possible to search for triatomines in wild ecotopes since their surveillance effort is restricted to residential homes; (iv) the bloodmeal source analysis was limited by the host blood controls selected for the molecular analysis reducing the host range detection. As a last limitation, the random sampling and small sample size could explain the low infestation levels and no identification of human transmission, further sampling including children evaluated during the certification process is required in order to detect incident cases.

## Conclusion

This investigation highlights the need to continue investigating *T. cruzi* transmission cycles propagated by native vector species in Miraflores, Colombia; a region certified free of intradomicile *R. prolixus-*mediated *T. cruzi* transmission. Despite historical public health efforts, secondary vector species have become an emerging challenge for contemporary Chagas disease control. Native secondary vectors were found infected in Miraflores inside and around the dwellings including *T. venosa, P. geniculatus,* and *P. rufotuberculatus.* The current seroprevalence rates found in humans were slightly higher than the national estimates, highlighting the need for assessment of a wider range of the population. Although no evidence of recent human transmission was identified, some areas were identified as high priority for public health intervention, due to high risk of active domestic transmission: Matarredonda Abajo, Pueblo y Cajón, Suna, and Morro Abajo. Evidence that triatomine vectors, domestic dogs and *D. marsupialis* are infected in the region highlights the need to redirect public health attention to assess human transmission risk. This study demonstrates that while historical triatomine vector control programs were successful in eliminating primary vectors, secondary vectors are emerging in their place and competent for contemporary disease transmission. Public health interventions to mitigate Chagas disease are still warranted today.

## Supporting information

S1 Table Variables significantly associated with number of triatomines collected inside the household. Shows the results of the Poisson regression for household characteristics and number of triatomines collected in each household that were statistically significant (p < 0.05).
(DOCX)

S2 Table Survey participant responses from household head compared to survey responses from households that provided human or canine sample. Describes the survey responses from each household head comparing the individuals that participated in providing biological samples and the total sampled households. No statistical comparison was made between the subset and the total sample.
(DOCX)

S3 Table Summary of infected and seropositive humans, dogs and triatomines by household and location. Shows the breakdown of samples collected per household that tested positive, or seropositive, and the location where these samples are coming from.
(DOCX)
