# Supplementary material for: Triatoma venosa and Panstrongylus geniculatus challenge the certification of interruption of vectorial Trypanosoma cruzi transmission by Rhodnius prolixus in eastern Colombia
Source: PLoS Negl Trop Dis. 2025 Jan 27;19(1):e0012822. doi: 10.1371/journal.pntd.0012822 (PMC11785281; doi:10.1371/journal.pntd.0012822)
Supplement: S1 Table — Shows the results of the Poisson regression for household characteristics and number of triatomines collected in each household that were statistically significant (p < 0.05). (DOCX) [file pntd.0012822.s001.docx]

**S1 Table . Variables associated with number of triatomines collected inside the household.**

| **Variables** | **Coefficient estimate** | **Confidence interval** | **P-value** |
| --- | --- | --- | --- |
| Time living in the household | -0.625 | (-0.05, -0.002) | 0.03 |
| **Wall material** |  |  |  |
| Adobe | 0.357 | (-0.51, 1.23) | 0.4 |
| Brick | 0.593 | (-0.58, 1.77) | 0.3 |
| Wood | -0.735 | (-1.77, 0.30) | 0.2 |
| Trodden Mud | -13.2 | (-1503, 1503) | 0.99 |
| Bahareque (Bamboo and soil) | -13.2 | (-1530, 1503) | 0.99 |
| **Wall finish** |  |  |  |
| Tarnished wall | 1.36 | (0.67 2.05) | <0.001 |
| Partially tarnished wall | -2.75 | (-4.73, -0.77) | 0.007 |
| Untarnished wall | 2.69 | (1.74, 3.63) | <0.001 |
| **Roof material** |  |  |  |
| Zinc | -2.24 | (-2.94, -1.53) | <0.001 |
| Mud tile | 3.23 | (2.57, 3.89) | <0.001 |
| Fiber cement tile | 3.16 | (1.18, 5.14) | 0.002 |
| Wood | 2.86 | (2.15, 3.57) | <0.001 |
| **Floor material** |  |  |  |
| Dirt floor | 1.97 | (1.26, 2.68) | <0.001 |
| Cement | 0.32 | (-0.55, 1.19) | 0.5 |
| Tile | -0.18 | (-0.96, 0.61) | 0.7 |
| Wood | 2.59 | (1.92, 3.25) | <0.001 |
| **Outdoor** **structure** |  |  |  |
| None | -14.3 | (-1458, 1430) | 0.99 |
| Chicken coop | 1.22 | (0.04, 2.40) | 0.04 |
| Barn | -1.72 | (-2.90, -0.54) | 0.004 |
| Stable | 1.16 | (0.12, 2.19) | 0.03 |
| Pigsty | -1.96 | (-3.94, 0.02) | 0.05 |
| Rabbit hutch | -1.05 | (-3.03, 0.93) | 0.3 |
| Porch | 3.23 | (2.57, 3.89) | <0.001 |
| Oven | 1.81 | (0.99, 2.63) | <0.001 |
| Wood pile | -0.89 | (-1.55, -0.22) | 0.009 |
| Rock pile | -0.13 | (-0.91, 0.66) | 0.7 |
| Presence of wattle^a^ | 2.31 | (1.58, 3.03) | <0.001 |
| Wall cracks | 1.96 | (1.14, 2.78) | <0.001 |
| Acceptable hygienic conditions | -0.40 | (-2.38, 1.58) | 0.7 |
| Number of lightbulbs inside the house | 0.11 | (-0.05, 0.26) | 0.2 |
| Number of lightbulbs around the house | 0.15 | (0.029, 0.27) | 0.01 |
| **Animal presence** |  |  |  |
| **Domestic** |  |  |  |
| Chickens | 2.79 | (0.81, 4.77) | 0.006 |
| Pigs | -1.31 | (-2.73, 0.11) | 0.07 |
| Cows | -0.89 | (-1.83, 0.05) | 0.06 |
| Horses | -1.05 | (-3.03, 0.93) | 0.3 |
| Cats | -1.64 | (-2.42, -0.86) | <0.001 |
| Fowl | -0.55 | (-2.53, 1.43) | 0.6 |
| Rabbits | 0.89 | (0.02, 1.77) | 0.004 |
| **Sylvatic** |  |  |  |
| Opossum | -1.05 | (-1.77, -0.32) | 0.005 |
| Rodents | 2.28 | (0.86, 3.70) | 0.002 |
| Bats | 19.0 | (-2385, 2423) | 0.99 |
| Wild rabbits | -13.2 | (-1530, 1503) | 0.99 |
| Presence inside the house^b^ | -15.3 | (-1573, 1542) | 0.99 |
| Presence around the house^b^ | 2.21 | (0.23 4.19) | 0.03 |
| Presence in the forest^b^ | -16.3 | (-1901, 1869) | 0.99 |
| **Vegetation around the house** |  |  |  |
| Palm trees | -13.2 | (-1530, 1503) | 0.99 |
| Bushes | -1.36 | (-2.03, -0.68.) | <0.001 |
| Trees | -1.36 | (-2.03, -0.68) | <0.001 |
| Epiphytic plants | -1.62 | (-2.44, -0.79) | <0.001 |
| **Type of environment surrounding the house** |  |  |  |
| Forest | 1.84 | (1.09, 2.59) | <0.001 |
| Pasture fields | -2.07 | (-3.49, -0.64) | 0.004 |
| Crop fields | 0.43 | (-0.75, 1.61) | 0.5 |

^a^Wattle refers to a structure that is outdoors under the roof as storage in rural areas to place the crops collected from the field.

^b^Sylvatic animals observed to be present inside or around the house.
