# Supplementary material for: Triatoma venosa and Panstrongylus geniculatus challenge the certification of interruption of vectorial Trypanosoma cruzi transmission by Rhodnius prolixus in eastern Colombia
Source: PLoS Negl Trop Dis. 2025 Jan 27;19(1):e0012822. doi: 10.1371/journal.pntd.0012822 (PMC11785281; doi:10.1371/journal.pntd.0012822)
Supplement: S2 Table — Describes the survey responses from each household head comparing the individuals that participated in providing biological samples and the total sampled households. No statistical comparison was made between the subset and the total sample. (DOCX) [file pntd.0012822.s002.docx]

**S2 Table.** **Survey participant responses from household head compared to survey responses from households that provided human or canine sample.**

| **Survey question description** | | **Household responses**  **N=118**  **N (%)^b^ or mean ± SD** | **Participating households**  **N=80**  **N (%)^b^ or mean ± SD** | **P-value^a^** |
| --- | --- | --- | --- | --- |
| How long have you lived in the household (mean ± SD) | | 23.4 ± 17.9 | 25.9 ± 17.9 |  |
| How many people live in your house (mean ± SD) | | 3.3 ± 2.0 | 3.5 ± 2.0 |  |
| **Entomological survey** | | |  |  |
| Triatomine presence | Have ever seen triatomines inside or around the household | 65 (55.1) | 47 (58.7) | 0.5 |
|  | Have seen triatomines inside or around the household in the past year | 22 (18.6) | 18 (22.5) | 0.07 |
| Triatomines seen inside the household | Kitchen | 1 (0.8) | 0 (0) | 0.3 |
|  | Bedroom | 13 (11.0) | 10 (12.5) | 0.8 |
|  | Living room | 7 (5.9) | 7 (8.7) | 1 |
|  | Dining room | 4 (3.4) | 3 (3.7) | 0.09 |
|  | Hallway | 6 (5.1) | 5 (6.2) | 0.2 |
|  | Bathroom | 5 (4.2) | 4 (5.0) | 1 |
|  | Entrance | 3 (2.5) | 3 (3.7) | 0.6 |
|  | Other | 1 (0.8) | 1 (1.2) | 1 |
| Triatomines seen outside the household | Chicken coop | 71 (60.2) | 49 (61.2) | 0.8 |
|  | Barn | 29 (24.6) | 22 (27.5) | 0.5 |
|  | Forest sighting | 51 (43.2) | 38 (47.5) | 0.3 |
|  | Pile of rocks | 39 (33.1) | 25 (31.2) | 0.7 |
|  | Pile of bricks | 13 (11.0) | 8 (10.0) | 0.5 |
|  | Pile of wood | 68 (57.6) | 46 (57.5) | 1 |
|  | Artificial light source | 64 (54.2) | 45 (56.2) | 0.6 |
|  | Bird nests | 3 (2.5) | 3 (3.7) | 0.6 |
|  | Palm trees | 5 (4.2) | 4 (5.0) | 1 |
|  | Cactus | 3 (2.5) | 2 (2.5) | 1 |
|  | Other | 9 (7.6) | 6 (7.5) | 1 |
| Triatomines seen in the outside perimeter | Crop fields | 79 (66.9) | 53 (66.2) | 0.8 |
|  | Forest | 65 (55.1) | 44 (55.0) | 1 |
| **Household characteristics** | | |  |  |
| Wall materials | Adobe | 15 (12.7) | 12 (15.0) | 0.6 |
|  | Brick | 102 (86.4) | 73 (91.2) | 0.02 |
|  | Wood | 24 (20.3) | 13 (16.2) | 0.08 |
|  | Trodden mud | 1 (0.8) | 1 (1.2) | 1 |
|  | Bamboo and soil (Bahareque) | 1 (0.8) | 0 (0) | 0.3 |
|  | Other | 6 (5.1) | 4 (5.0) | 1 |
| Wall finish | Completely tarnished | 40 (33.9) | 33 (41.2) | 0.03 |
|  | Partially tarnished | 39 (33.0) | 22 (33.7) | 0.8 |
|  | Not tarnished | 34 (28.8) | 20 (25.0) | 0.3 |
| Roof materials | Zinc | 93 (78.8) | 64 (80.0) | 0.6 |
|  | Mud tile | 6 (5.1) | 4 (5.0) | 1 |
|  | Fiber cement tile | 71 (60.2) | 53 (66.2) | 0.07 |
|  | Wood | 15 (12.7) | 12 (15.0) | 0.5 |
| Floor materials | Bare earth | 30 (25.4) | 21 (26.2) | 0.6 |
|  | Cement | 94 (76.7) | 65 (81.2) | 0.8 |
|  | Tiles | 27 (22.9) | 21 (26.1) | 0.3 |
|  | Wood | 12 (10.2) | 5 (6.2) | 0.04 |
|  | Other | 1 (0.8) | 0 (0) | 0.3 |
| Outdoor structures | None | 3 (2.5) | 2 (2.5) | 1 |
|  | Chicken coop | 92 (78.0) | 65 (81.2) | 0.2 |
|  | Barn | 36 (30.5) | 26 (32.5) | 0.5 |
|  | Manger | 4 (3.4) | 3 (3.7) | 1 |
|  | Pigsty | 20 (16.9) | 15 (18.7) | 0.8 |
|  | Hutch | 9 (7.6) | 5 (6.2) | 0.4 |
|  | Porch | 8 (6.8) | 7 (8.7) | 0.4 |
|  | Oven | 50 (42.4) | 38 (47.5) | 0.1 |
|  | Mill | 15 (12.7) | 9 (10.0) | 0.2 |
|  | Pile of wood | 70 (59.3) | 45 (56.2) | 0.5 |
|  | Pile of rocks | 26 (22.0) | 14 (17.5) | 0.3 |
|  | Other | 6 (5.1) | 6 (7.5) | 0.2 |
| Conditions | Presence of bushes | 26 (22.0) | 20 (25.0) | 0.5 |
|  | Presence of wall cracks | 47 (39.8) | 29 (36.2) | 0.3 |
|  | Acceptable hygienic conditions | 109 (92.4) | 74 (98.7) | 0.5 |
| Light bulbs inside the house (mean ± SD) | | 5.0 ± 1.8 | 5.2 ± 1.8 | 0.003 |
| Light bulbs outside the house (mean ± SD) | | 2.4 ± 1.7 | 2.5 ± 1.6 | 0.4 |
| **Presence of animals** | | |  |  |
| Domestic animals | Dogs | 102 (86.4) | 73 (91.2) | 0.02 |
|  | Chickens | 82 (69.5) | 60 (75.0) | 0.09 |
|  | Swine | 20 (16.9) | 17 (21.2) | 0.1 |
|  | Cattle | 32 (27.1) | 27 (33.7) | 0.04 |
|  | Horses | 8 (6.8) | 6 (7.5) | 1 |
|  | Cats | 70 (59.3) | 57 (71.2) | 0.001 |
|  | Domestic birds | 5 (4.2) | 3 (3.7) | 0.6 |
|  | Domestic rabbits | 9 (7.6) | 7 (9.7) | 0.7 |
| Sylvatic animals | None | 9 (7.6) | 6 (7.5) | 1 |
|  | Opossums | 59 (50.0) | 46 (56.2) | 0.03 |
|  | Rodents | 73 (61.9) | 54 (67.5) | 0.2 |
|  | Bats | 54 (45.8) | 38 (47.5) | 0.7 |
|  | Pigeons | 1 (0.8) | 1 (1.2) | 1 |
|  | Wild rabbits | 1 (0.8) | 1 (1.2) | 1 |
|  | Other | 20 (16.9) | 11 (13.7) | 1 |
| Sylvatic animal location | Inside the household | 8 (6.8) | 7 (8.7) | 0.4 |
|  | Around the household | 93 (78.8) | 66 (82.5) | 0.1 |
|  | In the forest | 13 (11.0) | 10 (12.5) | 0.7 |
| **Vegetation** **presence** | | |  |  |
| Type of vegetation | Palm trees | 1 (0.8) | 1 (1.2) | 1 |
|  | Bushes | 103 (87.3) | 70 (87.5) | 0.8 |
|  | Trees | 80 (67.8) | 59 (73.7) | 0.03 |
|  | Epiphyte plants | 63 (53.4) | 46 (57.5) | 0.4 |
|  | Forest | 39 (33.0) | 28 (35.0) | 0.5 |
|  | Pasture | 36 (30.5) | 22 (27.5) | 0.2 |
|  | Crop fields | 105 (89.0) | 72 (90.0) | 0.5 |

^a^P-value represents the statistically significant variables that have different proportion (Fisher’s exact test) or different mean value (t-test) between the individuals that were surveyed and the individuals that were sampled.

^b^Percentages may add more than 100% due to multiple choice options in the survey answers.
