# Supplementary material for: Triatoma venosa and Panstrongylus geniculatus challenge the certification of interruption of vectorial Trypanosoma cruzi transmission by Rhodnius prolixus in eastern Colombia
Source: PLoS Negl Trop Dis. 2025 Jan 27;19(1):e0012822. doi: 10.1371/journal.pntd.0012822 (PMC11785281; doi:10.1371/journal.pntd.0012822)
Supplement: S3 Table — Shows the breakdown of samples collected per household that tested positive, or seropositive, and the location where these samples are coming from. (DOCX) [file pntd.0012822.s003.docx]

**S3 Table. Summary of infected and seropositive humans, dogs and triatomines by household and location.**

| **Village** | **Seropositive humans** | **Humans evaluated*** | **Seropositive canines** | **Canines evaluated*** | **Canines PCR(+)** | **Positive/Collected Triatomines** | |
| --- | --- | --- | --- | --- | --- | --- | --- |
| Chapacía | 1 | 3 | 0 | 1 | 0 | - |  |
| Chapacía | 0 | 4 | 2 | 3 | 0 | - |  |
| Chapacía | 0 | 2 | - | - | - | 1/1 |  |
| Chapacía | 1 | 3 | - | - | - | - |  |
| Chapacía | 1 | 3 | 0 | 2 | 0 | - |  |
| Chapacía | 1 | 2 | - | - | - | - |  |
| Morro abajo | 1 | 2 | 0 | 1 | 0 | - |  |
| Morro abajo | 0 | 1 | 0 | 3 | 0 | 1/1 |  |
| Morro abajo | 1 | 2 | - | - | - | - |  |
| Morro abajo | 1 | 2 | - | - | - | - |  |
| Matarredonda abajo | 0 | 4 | 0 | 1 | 0 | 20/22 |  |
| Matarredonda abajo | 0 | 1 | - | - | - | 1/1 |  |
| Matarredonda abajo | 0 | 2 | 1 | 1 | 1 | - |  |
| Matarredonda abajo | 0 | 2 | 1 | 2 | 0 | - |  |
| Matarredonda abajo | 0 | 3 | 1 | 2 | 1 | - |  |
| Matarredonda abajo | - | - | 2 | 3 | 2 | - |  |
| Matarredonda abajo | 0 | 1 | 0 | 1 | 0 | - |  |
| Matarredonda abajo | 0 | 2 | 0 | 2 | 0 | - |  |
| Matarredonda abajo | 0 | 2 | 1 | 1 | 0 | - |  |
| Arrayan | 0 | 6 | - | - | - | 1/1 |  |
| Suna abajo | 1 | 2 | 0 | 1 | 0 | - |  |
| Suna abajo | 1 | 6 | - | - | - | 1/1 |  |
| Suna abajo | 0 | 4 | 1 | 4 | 1 | 3/3 |  |
| Suna abajo | 1 | 1 | 1 | 2 | 1 | - |  |
| Suna abajo | 0 | 4 | 0 | 1 | 0 | - |  |
| Suna abajo | 1 | 1 | - | - | - | - |  |
| Suna abajo | 0 | 1 | 1 | 2 | 1 | - |  |
| Suna abajo | 0 | 2 | - | - | - | 4/4 |  |
| Suna abajo | 1 | 3 | - | - | - | - |  |
| Suna abajo | - | - | 1 | 1 | 1 | - |  |
| Suna abajo | - | - | 1 | 2 | 0 | - |  |
| Pueblo y Cajón | 0 | 1 | - | - | - | 2/2 |  |
| Pueblo y Cajón | 1 | 2 | - | - | - | - |  |
| Pueblo y Cajón | 1 | 2 | - | - | - | - |  |

*Households with no seropositive dog or human are not included in this table.
